# Short-Term Associations Between Relative Humidity and Pediatric Afebrile Seizure Presentations: A Distributed Lag Non-Linear Time-Series Study

**DOI:** 10.3390/children13091251

**Published:** 2026-09-15

**Authors:** Fatih Battal, Gizem Demirtas, Zahide Acar

**Affiliations:** 1 Department of Pediatrics, Faculty of Medicine, Canakkale Onsekiz Mart University, Canakkale 17020, Türkiye; 2 Department of Geography, Faculty of Humanities and Social Sciences, Canakkale Onsekiz Mart University, Canakkale 17100, Türkiye

**Keywords:** child, emergency service, hospital, humidity, temperature, seizures

## Abstract

**Highlights:**

**What are the main findings?**

- Relative humidity was the strongest meteorological predictor of pediatric afebrile seizure presentations.
- A 10-percentage-point increase in relative humidity was associated with an 18.6% higher rate of pediatric afebrile seizure presentations.
- The humidity-associated increase in afebrile seizure presentation rates persisted across a 0–3-day lag period.

**What are the implications of the main findings?**

- Temperature and precipitation showed less consistent associations across analytical models.
- Distributed lag modeling identified both immediate and delayed humidity-related effects.

**Abstract:**

Objective: To evaluate the association between daily meteorological variability and pediatric afebrile seizure presentations using ecological time-series analysis and Distributed Lag Non-linear Models (DLNMs). Methods: This retrospective ecological time-series study was conducted in a tertiary pediatric emergency department in northwestern Türkiye. Pediatric seizure-related presentations recorded between January 2019 and December 2023 were retrospectively screened for case identification and clinical validation. The primary environmental exposure analysis was restricted to eligible afebrile seizure presentations occurring during the 2023 calendar year and linked to synchronized daily meteorological data. Associations between meteorological variables and daily afebrile seizure presentation counts were assessed using multivariable Quasi-Poisson regression and Distributed Lag Non-linear Models (DLNMs). Results: Among 777 screened seizure-related presentations, 67 children contributing 157 afebrile seizure presentations during 2023 were included. Relative humidity showed the strongest independent association with pediatric afebrile seizure presentations. Each 10-percentage-point increase in relative humidity was associated with an 18.6% higher rate of pediatric afebrile seizure presentations (IRR = 1.186; 95% CI: 1.103–1.275; *p* < 0.001). DLNM analysis demonstrated a significant cumulative delayed association, with a 10-percentage-point increase in relative humidity associated with a 15% higher cumulative rate of afebrile seizure presentations across the 0–3-day lag period (cumulative IRR = 1.15; 95% CI: 1.08–1.22; *p* < 0.001). Ambient temperature and precipitation demonstrated less consistent associations across analytical approaches, whereas humidity-related effects remained robust throughout all analyses. Conclusions: Higher relative humidity was associated with increased pediatric afebrile seizure presentations and represented the only meteorological exposure demonstrating both significant contemporaneous and cumulative delayed associations. These findings suggest that meteorological variability, particularly relative humidity, may contribute to short-term and delayed fluctuations in seizure-related healthcare utilization among children. Given the single-center ecological design and the one-year environmental exposure period, these findings should be interpreted as population-level and hypothesis-generating and should not be considered a basis for individual risk prediction or humidity-based clinical counseling. Further multicenter, multi-year prospective studies incorporating individual-level and higher-resolution environmental exposure assessment are needed to confirm these findings and clarify the biological mechanisms underlying humidity-associated seizure susceptibility in pediatric populations.

## 1. Introduction

Environmental and meteorological conditions are increasingly recognized as important determinants of human health. Short-term fluctuations in weather conditions have been associated with a variety of acute health outcomes, and children are considered particularly vulnerable to environmental stressors because of developmental differences in thermoregulation, fluid balance, metabolic reserve, and neurophysiological adaptation mechanisms [1,2,3,4]. Consequently, variations in ambient temperature, humidity, and other atmospheric conditions may influence pediatric neurological health.

Seizures are among the most common pediatric neurological emergencies and account for a substantial proportion of pediatric emergency department visits [5,6]. Although febrile seizures constitute a large proportion of seizure-related presentations in early childhood, afebrile seizures are clinically more heterogeneous and may reflect underlying epileptic syndromes, structural abnormalities, metabolic disturbances, or idiopathic neuronal hyperexcitability [7]. The lifetime risk of experiencing an afebrile seizure is approximately 1%, and recurrent seizures remain clinically important because of their association with long-term neurological morbidity [8]. Despite advances in pediatric neurology, the underlying cause of epilepsy remains unidentified in a considerable proportion of patients, suggesting that environmental factors may contribute to seizure susceptibility.

Meteorological variability has been investigated as a potential contributor to adverse health outcomes, including asthma exacerbations, migraine attacks, cerebrovascular disorders, and cardiovascular events [9,10,11,12,13]. However, studies evaluating the relationship between meteorological conditions and seizure occurrence have produced inconsistent findings. While some investigations have reported associations between seizure frequency and atmospheric pressure, temperature, or humidity, others have failed to identify significant relationships [14,15,16]. These discrepancies may reflect differences in climatic characteristics, exposure assessment methods, study populations, and analytical approaches.

Several biological mechanisms have been proposed to explain potential meteorological influences on seizure susceptibility. Temperature-sensitive ion channels, particularly members of the transient receptor potential (TRP) family, have been implicated in the regulation of neuronal excitability under varying thermal conditions [17,18,19]. In addition, humidity-related alterations in thermal perception and thermoregulatory efficiency may contribute to physiological stress responses, sleep disturbance, autonomic dysregulation, and altered neural homeostasis, all of which have been suggested as factors capable of lowering seizure thresholds [20,21,22,23,24,25]. Although these mechanisms remain incompletely understood, they provide biologically plausible pathways linking environmental conditions to seizure occurrence.

Growing concern regarding climate variability and increasing environmental extremes has intensified interest in the neurological consequences of weather-related exposures [26,27,28]. Nevertheless, pediatric data evaluating the relationship between meteorological conditions and afebrile seizure presentations remain limited, particularly in coastal Mediterranean regions characterized by substantial humidity variability [21]. Moreover, most previous studies have focused on adult populations or established epilepsy cohorts rather than clinically validated pediatric afebrile seizure presentations [14,15,16,29,30,31]. Importantly, few studies have examined meteorological influences on pediatric afebrile seizure presentations using validated emergency department cohorts, and even fewer have evaluated both immediate and delayed exposure effects within a unified analytical framework. In addition, few studies have applied contemporary time-series methods capable of capturing complex exposure–lag relationships and potential delayed meteorological effects [32].

Addressing these gaps is clinically relevant because meteorological factors may contribute to short-term fluctuations in seizure-related healthcare utilization and could identify potentially vulnerable periods for children with seizure disorders. Improved understanding of weather–seizure relationships may contribute to a better characterization of short-term environmental influences on seizure-related healthcare utilization and may inform future research and healthcare resource planning.

Therefore, this study aimed to evaluate the association between daily meteorological variables and pediatric afebrile seizure presentations in a tertiary pediatric emergency department. Using a Distributed Lag Non-linear Modeling (DLNM) framework, we investigated whether short-term variations in relative humidity, ambient temperature, and precipitation were associated with immediate and delayed changes in pediatric afebrile seizure presentations after adjustment for temporal and seasonal confounders [32]. To minimize the influence of pandemic-related disruptions in healthcare utilization, the primary environmental exposure analyses were restricted to the 2023 calendar year, during which complete synchronized daily meteorological data were available.

## 2. Materials and Methods

### 2.1. Study Design and Setting

This retrospective ecological time-series study was conducted at the Pediatric Emergency Department of Çanakkale Onsekiz Mart University Faculty of Medicine, a tertiary referral center in northwestern Türkiye. The study was approved by the institutional ethics committee (Decision No: 2024/06-01).

Pediatric seizure-related emergency department presentations recorded between 1 January 2019 and 31 December 2023 were screened to identify afebrile seizure cases.

This five-year period was used exclusively for case identification and clinical screening and did not constitute the environmental exposure analysis period. The primary ecological time-series analysis evaluating associations between meteorological exposures and daily afebrile seizure presentations was restricted to the 2023 calendar year.

Daily meteorological measurements were linked to daily counts of eligible pediatric afebrile seizure presentations occurring between 1 January and 31 December 2023. The 2023 calendar year was selected as the predefined environmental exposure period because complete and continuously synchronized daily meteorological data were available throughout this period. In addition, healthcare utilization patterns during 2019–2022 were substantially influenced by the COVID-19 pandemic and associated public health measures, potentially affecting seizure-related emergency department presentations. Restricting the primary analysis to 2023 therefore provided a more temporally homogeneous dataset for evaluating short-term meteorological associations. Accordingly, all meteorological exposure–outcome analyses, including the Quasi-Poisson regression and DLNM analyses, were based exclusively on the synchronized 2023 clinical and meteorological dataset.

### 2.2. Patient Selection and Eligibility Criteria

Patients aged 1 month to 18 years presenting with afebrile seizures were eligible for inclusion. Afebrile seizures were defined according to International League Against Epilepsy (ILAE) criteria as paroxysmal motor, sensory, behavioral, or consciousness-related events occurring in the absence of fever (body temperature < 38.0 °C).

Potential cases were initially identified using seizure-related International Classification of Diseases (ICD) diagnostic codes and subsequently validated through manual review of emergency department records, pediatric neurology consultations, laboratory findings, neuroimaging reports when available, and electroencephalographic evaluations.

Exclusion criteria included neonates aged ≤30 days, febrile seizures, seizures secondary to acute metabolic disturbances, trauma, intracranial infections, toxic exposures, documented medication non-adherence, psychogenic non-epileptic events, and refractory epilepsy syndromes. Patients with documented fever or antipyretic exposure within the preceding 24 h were also excluded.

Several identifiable acute conditions capable of precipitating seizures were addressed through the predefined exclusion criteria. These criteria were intended to reduce the potential influence of major identifiable acute seizure precipitants that could independently contribute to afebrile seizure presentations and potentially confound the observed associations with meteorological exposures. In particular, seizures attributable to acute metabolic disturbances, intracranial infections, trauma, toxic exposures, documented medication non-adherence, or recent febrile illness were excluded from the analytic cohort.

Following validation, 67 unique pediatric patients accounting for 157 emergency department presentations during 2023 were included in the final analysis.

### 2.3. Outcome Definition

The primary outcome was the daily number of pediatric afebrile seizure presentations to the emergency department.

Because the analytical unit was the daily event count rather than the individual patient, recurrent presentations by the same patient were retained in accordance with ecological time-series methodology. Daily seizure counts were calculated as the total number of eligible afebrile seizure-related emergency department visits recorded on each calendar day during the study period.

For analyses according to seizure history, the index emergency department presentation was defined as the first eligible afebrile seizure-related presentation of each patient occurring during the 2023 environmental exposure analysis period. Seizure history was determined through review of the available electronic medical records, including previous emergency department and outpatient records, pediatric neurology documentation, and the clinical history recorded at the index presentation. Established epilepsy was defined as a documented diagnosis of epilepsy preceding the index presentation. First-time afebrile seizure presentation was defined as an eligible afebrile seizure presentation in a child for whom no previous unprovoked/afebrile seizure or established epilepsy was documented in the available medical record or clinical history.

No fixed-duration lookback window was imposed for ascertainment of previous seizure history or epilepsy. All available historical information preceding the index presentation in the institutional electronic medical record, together with the clinical history documented at presentation, was reviewed. Consequently, the effective lookback period varied according to the duration and completeness of the available medical record for each patient.

### 2.4. Meteorological Data Collection

Meteorological data were obtained from the Turkish State Meteorological Service (TSMS). Daily measurements were collected from the regional monitoring station serving the study area and recorded as standardized 24-h averages. The monitoring station represented the official regional reference station for the study catchment area and provided continuous meteorological measurements throughout the study period.

Meteorological variables evaluated included:

- Mean ambient temperature (°C).
- Relative humidity (%).
- Atmospheric pressure (hPa).
- Wind speed (m/s).
- Precipitation (mm).

Daily meteorological measurements were synchronized with emergency department presentation dates to construct exposure–response datasets. Environmental exposures were assessed at the population level, consistent with the ecological study design.

Meteorological exposures were assigned according to the calendar date of each emergency department presentation using standardized 24-h daily averages. Accordingly, for presentations occurring earlier in the day, the same-day (lag 0) exposure metric could include meteorological observations recorded after seizure onset or emergency department presentation. Because hourly meteorological measurements were not available for the present analysis, exposure windows could not be aligned to the exact time of seizure onset or emergency department presentation. This exposure-assignment approach was applied consistently across all study days and reflects the daily temporal resolution of the ecological time-series design.

### 2.5. Statistical Analysis

Descriptive statistics were calculated for demographic, clinical, and meteorological variables. Continuous variables were summarized as mean ± standard deviation (SD) or median with interquartile range (IQR), as appropriate, whereas categorical variables were reported as frequencies and percentages.

Spearman rank correlation analyses were initially performed to descriptively explore associations between individual meteorological variables and daily afebrile seizure presentation counts. Correlation analyses were considered exploratory and were not used as a formal variable-selection procedure or to exclude meteorological predictors from the multivariable models. All prespecified meteorological variables—mean ambient temperature, relative humidity, atmospheric pressure, wind speed, and precipitation—were retained in the primary multivariable model irrespective of the magnitude or statistical significance of their univariable correlations.

Comparisons between days with and without afebrile seizure presentations were performed using Student’s *t*-test or the Mann–Whitney U test, as appropriate according to the distribution of the data. These analyses were considered exploratory and descriptive.

For relative humidity, effect estimates were expressed per 10-percentage-point increase in relative humidity (e.g., from 50% to 60%), rather than per 10% relative increase. Throughout the manuscript, IRRs were interpreted as relative changes in the rate of pediatric afebrile seizure presentations rather than as changes in population-level seizure incidence.

Because the primary outcome consisted of daily count data with evidence of mild overdispersion, Quasi-Poisson regression was used as the primary analytical approach. Preliminary model diagnostics demonstrated modest overdispersion, supporting the use of Quasi-Poisson rather than standard Poisson regression. Incidence rate ratios (IRRs) and corresponding 95% confidence intervals (CIs) were estimated to quantify relative changes in the daily rate of pediatric afebrile seizure presentations associated with predefined increments in meteorological exposures.

The multivariable model included mean ambient temperature, relative humidity, atmospheric pressure, wind speed, precipitation, and a binary weekend indicator. Weekend was defined as Saturday or Sunday, with weekdays serving as the reference category. Seasonality and longer-term temporal variation within the 2023 study period were jointly controlled by modeling calendar time as a continuous variable using a natural cubic spline with 4 degrees of freedom.

Distributed Lag Non-linear Models (DLNMs) were subsequently fitted using the dlnm package in R to characterize potential nonlinear and delayed associations between meteorological exposures and daily pediatric afebrile seizure presentations. For relative humidity, the exposure–response dimension of the cross-basis function was modeled using a natural cubic spline with 3 degrees of freedom, with two internal knots placed at the 33.3rd and 66.7th percentiles of the observed relative humidity distribution. The lag–response dimension was modeled using a natural cubic spline with 3 degrees of freedom across the predefined 0–3-day lag window, corresponding to one internal knot at the median of the lag distribution (lag 1.5 days). Relative humidity was centered at the median observed value (63%).

A maximum lag of 3 days was prespecified to capture short-term and potentially delayed meteorological effects while maintaining a parsimonious model appropriate for the one-year daily time series and the observed number of events. This lag window was considered biologically plausible because short-term meteorological exposures may influence seizure susceptibility through transient changes in thermoregulation, autonomic function, sleep, and physiological homeostasis, with such effects expected to occur over a relatively short time scale. The adequacy of the selected lag structure was further evaluated using residual partial autocorrelation function (PACF) analyses. Residual PACF analyses were additionally examined to assess whether meaningful temporal dependence remained after model fitting. No substantial residual autocorrelation was observed beyond the short-term lag structure considered in the primary model.

To assess whether the observed association was sensitive to the choice of maximum lag, additional sensitivity analyses were performed by extending the DLNM lag window to 0–5 and 0–7 days while retaining the same exposure–response and temporal adjustment specifications. The direction and overall pattern of the humidity-associated estimates remained consistent across these extended lag specifications, supporting the robustness of the primary 0–3-day model.

Exploratory subgroup analyses were performed according to age group, season, and seizure history. Children were categorized as preschool-aged (<6 years) or school-aged (≥6 years). The warm season was defined as April–September and the cold season as October–March. Seizure-history categories were defined as described in Section 2.3.

Multicollinearity among meteorological variables was assessed using variance inflation factors (VIFs), with values > 5 considered indicative of potentially problematic multicollinearity. Heatwave days were defined as days with mean ambient temperatures exceeding the 95th percentile of the annual temperature distribution.

Sensitivity analyses were performed to evaluate the robustness of the primary findings. Alternative model specifications included standard Poisson regression, negative binomial regression, univariate humidity models, and models additionally adjusted for heatwave conditions. Sensitivity analyses focused on relative humidity because it demonstrated the strongest and most consistent association across the exploratory analyses and primary multivariable models. In addition, to evaluate whether the primary findings were disproportionately influenced by a small number of children with recurrent emergency department presentations, the distribution of presentations per patient was examined. Frequent presenters were defined as patients with ≥5 eligible afebrile seizure presentations during 2023, and the primary relative-humidity model was refitted after excluding presentations contributed by these patients.

Model goodness-of-fit was evaluated using deviance statistics, Pearson χ^2^ statistics, residual degrees of freedom, deviance-to-degrees-of-freedom ratios, and overdispersion parameters. Residual temporal autocorrelation was assessed graphically using PACF plots.

All statistical tests were two-sided, and a *p*-value < 0.05 was considered statistically significant. All statistical analyses were performed using R statistical software version 4.6.1 (R Foundation for Statistical Computing, Vienna, Austria).

## 3. Results

### 3.1. Study Population and Event Characteristics

**Figure 1:**
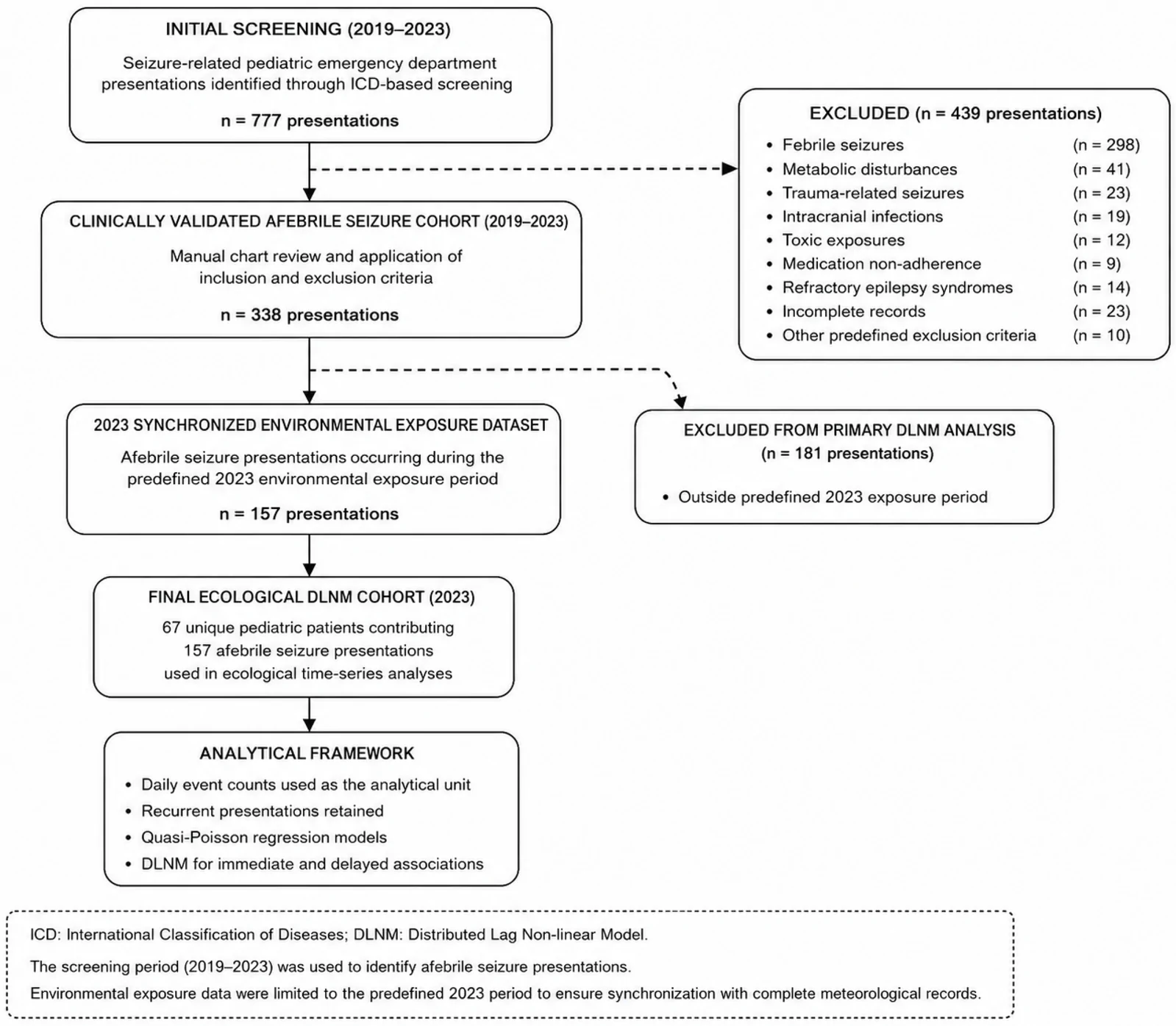
Flow diagram illustrating patient selection and construction of the ecological time-series cohort. Among 777 seizure-related pediatric emergency department presentations screened between January 2019 and December 2023, 338 clinically validated afebrile seizure presentations fulfilled eligibility criteria after manual chart review and application of predefined exclusion criteria. Of these, 157 presentations occurring during the predefined 2023 environmental exposure period were included in the synchronized ecological dataset. The final ecological DLNM cohort consisted of 67 unique pediatric patients contributing 157 afebrile seizure presentations that were analyzed as daily event counts within the ecological time-series framework. Dashed arrows indicate the exclusion pathway during patient selection.

A total of 67 pediatric patients met the predefined eligibility criteria and were included in the final ecological analysis (Figure 1). The mean age was 6.23 ± 4.5 years (median 5.3 years; range 2 months–17 years), and 47.9% were female (*n* = 32).

These 67 unique patients contributed a total of 157 afebrile seizure-related emergency department presentations during the 2023 study period. Because the unit of analysis was daily seizure occurrence, recurrent presentations from the same patient were retained in the ecological time-series framework.

The distribution of presentations per patient was additionally examined to determine whether the overall event count was disproportionately influenced by a small number of children with recurrent presentations. Of the 67 unique patients, 27 (40.3%) contributed one eligible presentation, 19 (28.4%) contributed two presentations, 13 (19.4%) contributed three to four presentations, and 8 (11.9%) contributed ≥5 presentations during 2023. Patients with ≥5 presentations accounted for 47 of the 157 eligible presentations (29.9%).

Among the included patients, 39 (58.2%) were preschool-aged (<6 years) and 28 (41.8%) were school-aged (≥6 years). Forty patients (59.7%) had a previously established diagnosis of epilepsy, whereas 27 (40.3%) were classified as having a first-time afebrile seizure presentation according to the predefined seizure-history criteria.

During the study period, the mean daily seizure frequency was 0.43 events/day (median 0; IQR 0–1), with a maximum of four presentations recorded on a single day. Seizure presentations occurred on 102 days (27.9%) during the study year, whereas no seizure-related presentations were recorded on the remaining 263 days (72.1%).

Descriptive statistics for daily meteorological variables and seizure presentations are summarized in Table 1.

### 3.2. Exploratory Analyses of Meteorological Variables

Exploratory Spearman correlation analyses demonstrated a positive correlation between relative humidity and daily pediatric afebrile seizure presentation counts and an inverse correlation with ambient temperature (Supplementary Table S1). Relative humidity showed the strongest univariable correlation with daily afebrile seizure presentation counts (ρ = 0.32, *p* < 0.001), whereas ambient temperature showed a modest inverse correlation (ρ = −0.21, *p* < 0.01). These correlations were interpreted descriptively and were not used to select or exclude predictors from the primary multivariable model.

Comparisons between seizure days and seizure-free days demonstrated significantly higher relative humidity (69.2% vs. 64.1%, *p* < 0.001) and lower ambient temperature (16.8 °C vs. 19.1 °C, *p* = 0.041) on days with seizure presentations. No significant differences were observed for atmospheric pressure, wind speed, or precipitation (Supplementary Table S2).

### 3.3. Multivariable Regression Analysis

In multivariable Quasi-Poisson regression models adjusted for seasonality, long-term temporal trends, and day-of-week effects, relative humidity remained independently associated with pediatric afebrile seizure presentations. Each 10-percentage-point increase in relative humidity was associated with an 18.6% higher rate of daily pediatric afebrile seizure presentations (IRR = 1.186; 95% CI: 1.103–1.275; *p* < 0.001).

**Figure 2:**
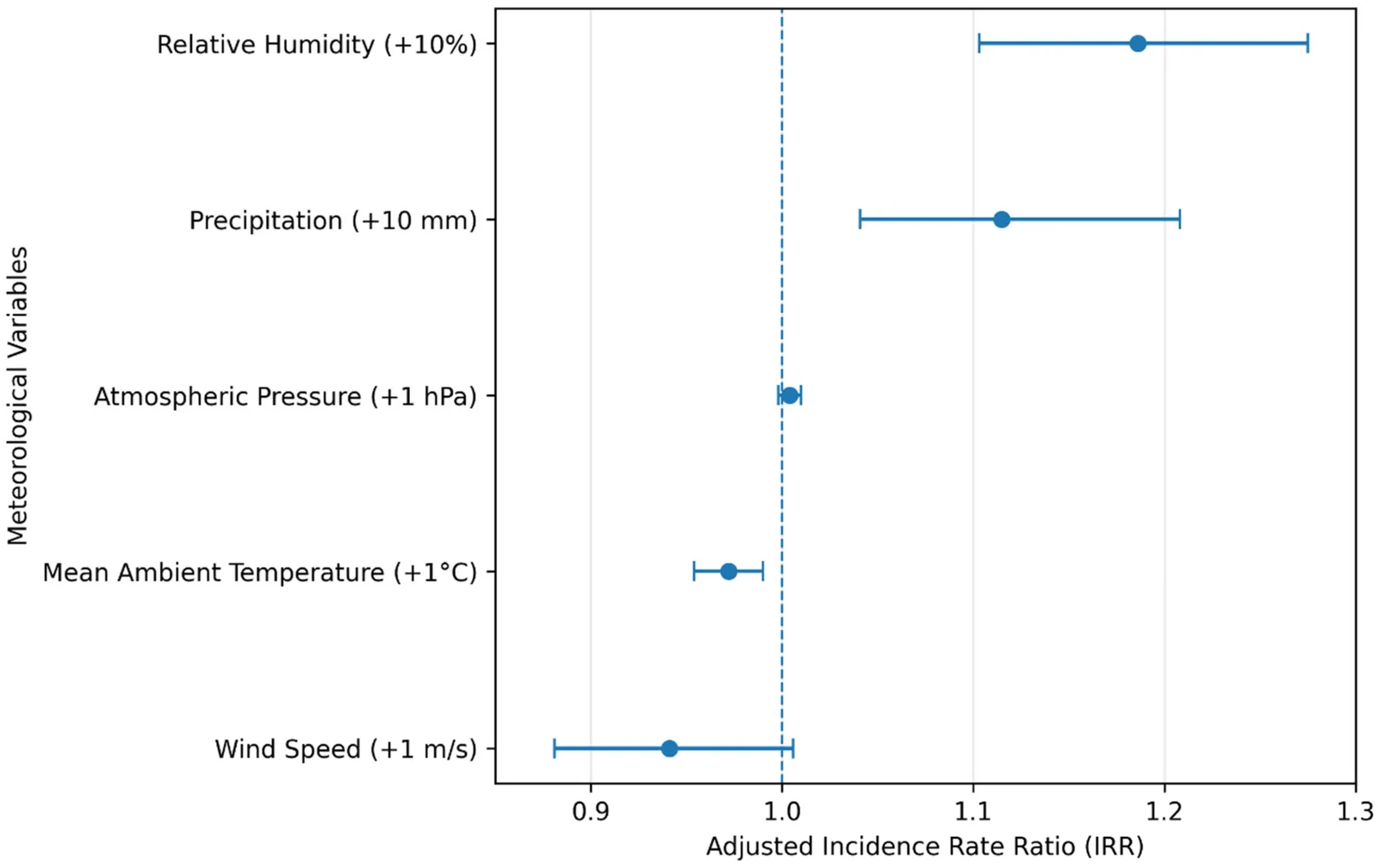
Multivariable Associations Between Meteorological Variables and Daily Pediatric Afebrile Seizure Presentations. Forest plot illustrating adjusted incidence rate ratios (IRRs) and corresponding 95% confidence intervals derived from the final multivariable Quasi-Poisson regression model. Relative humidity demonstrated the strongest positive association with pediatric afebrile seizure presentations, with each 10-percentage-point increase associated with an 18.6% higher daily presentation rate. Higher ambient temperature was associated with a lower daily presentation rate, whereas precipitation demonstrated a modest positive association. Atmospheric pressure and wind speed were not significantly associated with seizure presentations. The vertical dashed line indicates an IRR of 1.0 (no association). Models were adjusted for seasonality, long-term temporal trends, and day-of-week effects. Abbreviations: IRR, incidence rate ratio; CI, confidence interval.

Higher ambient temperature was independently associated with a lower rate of daily pediatric afebrile seizure presentations (IRR = 0.972; 95% CI: 0.954–0.990; *p* = 0.003). Precipitation was also positively associated with the daily presentation rate, with each 10-mm increase associated with an 11.5% higher rate of pediatric afebrile seizure presentations (IRR = 1.115; 95% CI: 1.041–1.208; *p* = 0.002)**.** Atmospheric pressure and wind speed were not significantly associated with pediatric afebrile seizure presentations (Table 2; Figure 2).

Among all evaluated meteorological variables, relative humidity exhibited the largest positive effect estimate in the multivariable models.

### 3.4. Distributed Lag Non-Linear Model Findings

**Figure 3:**
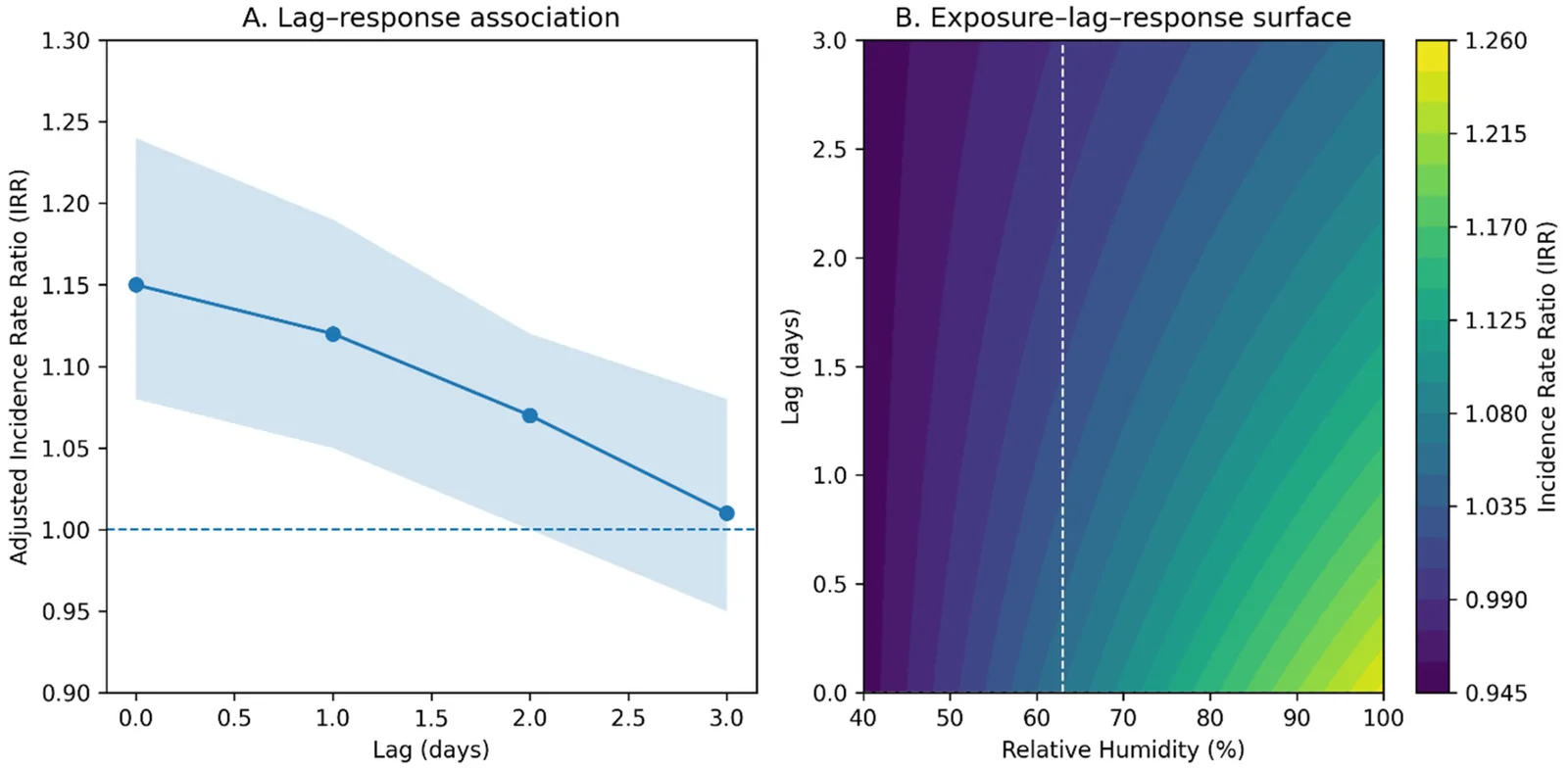
Distributed Lag Non-linear Model (DLNM) Analysis of Relative Humidity and Pediatric Afebrile Seizure Presentations. (**A**) Lag–response curve illustrating the association between relative humidity and pediatric afebrile seizure presentations across the predefined 0–3-day lag period. Solid lines represent adjusted incidence rate ratios (IRRs), and shaded areas indicate 95% confidence intervals. (**B**) Exposure–lag–response surface demonstrating the combined association of relative humidity and lag time with pediatric afebrile seizure presentations. Higher humidity levels were associated with higher presentation-rate estimates, particularly during shorter lag periods, whereas the estimates progressively attenuated across subsequent lag intervals. The reference humidity value was set at the median relative humidity observed during the study period (63%). The horizontal dashed line in panel (**A**) indicates an IRR of 1.0 (no association), whereas the vertical dashed line in panel (**B**) indicates the reference relative humidity value of 63%. Models were adjusted for seasonality, long-term temporal trends, and day-of-week effects. Abbreviations: DLNM, Distributed Lag Non-linear Model; IRR, incidence rate ratio; CI, confidence interval.

Distributed Lag Non-linear Model analyses demonstrated a significant cumulative association between relative humidity and pediatric afebrile seizure presentations. Across the predefined 0–3-day lag period, a 10-percentage-point increase in relative humidity was associated with a 15% higher cumulative rate of pediatric afebrile seizure presentations (cumulative IRR = 1.15; 95% CI: 1.08–1.22; *p* < 0.001). (Table 3; Figure 3).

Lag-specific estimates indicated that the humidity-associated rate was highest at lag 0 (IRR = 1.07; 95% CI: 1.02–1.12) and remained elevated at lag 1 (IRR = 1.05; 95% CI: 1.01–1.10), followed by progressive attenuation at lag 2 (IRR = 1.03; 95% CI: 0.99–1.08) and lag 3 (IRR = 1.01; 95% CI: 0.97–1.06). Thus, the observed association was concentrated predominantly within the first 1–2 days after exposure (Figure 3A). The exposure–lag–response surface showed a corresponding pattern, with higher relative humidity levels associated with higher rate estimates, particularly at shorter lags. The magnitude of the association progressively attenuated with increasing lag time and approached the null by lag 3 (Figure 3B). Relative humidity was centered at the median observed value of 63%.

**Figure 4:**
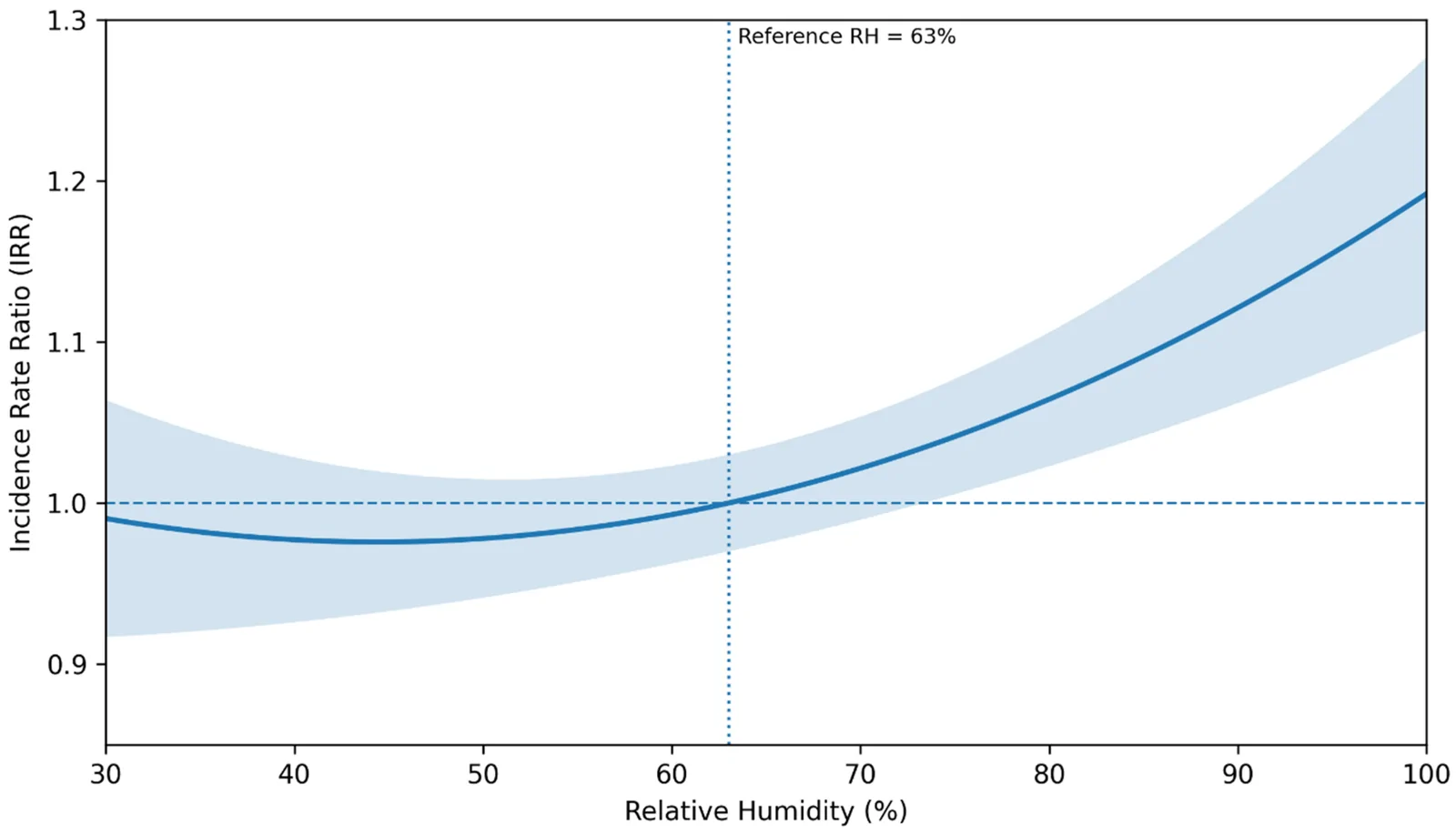
Exposure–Response Relationship Between Relative Humidity and Pediatric Afebrile Seizure Presentations. The horizontal dashed line indicates an IRR of 1.0 (no association), whereas the vertical dashed line indicates the reference relative humidity value of 63%. Abbreviations: IRR, incidence rate ratio; CI, confidence interval.

The exposure–response curve demonstrated a nonlinear positive association between relative humidity and pediatric afebrile seizure presentations, with the median relative humidity of 63% used as the reference (IRR = 1.00). Relative to this reference, estimated rate ratios increased progressively at higher humidity levels, reaching approximately 1.06 (95% CI: 1.02–1.11) at 80% relative humidity, 1.12 (95% CI: 1.06–1.18) at 90%, and 1.19 (95% CI: 1.11–1.28) at 100% (Figure 4). At lower relative humidity levels, rate estimates remained close to or slightly below the reference, with confidence intervals generally including unity. Overall, the exposure–response pattern indicated that the positive association became more pronounced at higher relative humidity levels.

In contrast, the cumulative delayed effect of ambient temperature across the same lag period was not statistically significant (IRR = 0.98; 95% CI: 0.91–1.06; *p* = 0.410). Similarly, precipitation did not demonstrate a statistically significant cumulative delayed association across the 0–3-day lag period (IRR = 1.03; 95% CI: 0.98–1.09; *p* = 0.214).

### 3.5. Sensitivity, Subgroup, and Diagnostic Analyses

Sensitivity analyses using alternative statistical specifications yielded stable humidity-associated presentation-rate estimates across all modeling strategies (IRR range: 1.14–1.18), supporting the robustness of the primary findings (Supplementary Table S3).

Additional sensitivity analyses extending the maximum DLNM lag window from the primary 0–3-day specification to 0–5 and 0–7 days yielded humidity-associated estimates that remained similar in direction and overall pattern. These findings indicated that the observed association was not materially dependent on the prespecified 3-day maximum lag.

Subgroup analyses demonstrated stronger humidity-associated presentation-rate estimates among preschool children (IRR = 1.14; 95% CI: 1.06–1.23), during the warm season (IRR = 1.17; 95% CI: 1.08–1.27), and among patients with established epilepsy (IRR = 1.15; 95% CI: 1.07–1.24) (Supplementary Table S4). The highest point estimate was observed during the warm season.

In contrast, weaker and statistically non-significant associations were observed during the cold season (IRR = 1.05; 95% CI: 0.98–1.13) and among patients classified as having a first-time afebrile seizure presentation (IRR = 1.04; 95% CI: 0.97–1.12). Although these exploratory analyses were not designed to formally compare effect sizes between subgroups, the overall direction of association remained consistent across demographic and clinical categories.

To evaluate whether recurrent presentations from a small number of children disproportionately influenced the primary findings, the relative-humidity model was refitted after excluding patients meeting the predefined frequent-presenter criterion (≥5 eligible presentations during 2023). After exclusion of these patients, the association between relative humidity and daily pediatric afebrile seizure presentations remained similar in direction and magnitude to that observed in the primary analysis (IRR per 10-percentage-point increase in relative humidity = 1.17; 95% CI: 1.07–1.28; *p* < 0.001). This finding suggested that the primary humidity association was not driven predominantly by a small subgroup of children with frequent recurrent presentations.

Model diagnostic analyses demonstrated acceptable model fit and modest overdispersion (φ = 1.12; Supplementary Table S5). Variance inflation factor values ranged from 1.10 to 1.47, with all values below the prespecified threshold of 5, indicating no evidence of problematic multicollinearity among the meteorological predictors (Supplementary Table S6).

Residual diagnostics demonstrated no evidence of significant temporal autocorrelation across lags 1–20, supporting the adequacy of the final modeling framework and the validity of model assumptions.

Overall, diagnostic and sensitivity analyses supported the robustness of the observed association between relative humidity and pediatric afebrile seizure presentations. Subgroup findings were considered exploratory and should not be interpreted as evidence of statistically established effect modification.

## 4. Discussion

This study evaluated the association between meteorological variability and pediatric afebrile seizure presentations in a coastal Mediterranean region using ecological time-series methods and Distributed Lag Non-linear Models (DLNMs). The principal finding was that increased relative humidity was consistently associated with a higher rate of pediatric afebrile seizure presentations. Relative humidity emerged as the strongest independent meteorological predictor across the analytical approaches, with each 10-percentage-point increase associated with an 18.6% higher rate of daily afebrile seizure presentations and a 15% higher cumulative presentation rate across the 0–3-day lag period. These associations remained stable across multiple sensitivity analyses. In contrast, ambient temperature and precipitation demonstrated less consistent findings. Collectively, these results suggest that atmospheric humidity may represent an environmental factor associated with short-term fluctuations in seizure-related healthcare utilization among children.

Previous studies investigating meteorological influences on seizure occurrence have produced heterogeneous findings across geographic regions and climatic settings [14,15,29,31,33,34]. While some studies reported increased seizure occurrence during colder periods, others identified associations with elevated humidity or abrupt weather changes [13,14,15,29,31]. The present findings align more closely with studies suggesting a role for atmospheric moisture in seizure occurrence. The observed variability among studies may reflect differences in climate characteristics, exposure assessment methods, study populations, seizure definitions, exposure metrics, and analytical methodologies. Our findings further support the concept that local climatic conditions may influence the nature and magnitude of observed weather–seizure associations.

To our knowledge, few pediatric studies have simultaneously evaluated immediate and delayed meteorological associations with afebrile seizure presentations using a DLNM framework. By integrating ecological time-series analyses with distributed lag modeling, the present study provides evidence that associations between atmospheric humidity and seizure-related healthcare utilization may occur both on the day of exposure and across subsequent days. These findings extend previous weather–seizure literature by demonstrating a short-term lag pattern in a pediatric population.

An important observation was the delayed exposure pattern identified through DLNM analysis. Humidity-associated presentation-rate estimates were highest during the early lag period and progressively attenuated thereafter. This pattern suggests that the association between meteorological conditions and seizure-related presentations may extend beyond same-day exposure. The identification of delayed associations highlights the value of distributed lag modeling when evaluating weather-related neurological outcomes and may partially explain inconsistencies among studies relying solely on same-day exposure analyses.

An additional noteworthy finding was the contrasting pattern observed for temperature and humidity. Although ambient temperature demonstrated an inverse association with seizure presentations in contemporaneous multivariable analyses, this effect was not retained in distributed lag models. In contrast, relative humidity demonstrated both a significant contemporaneous association and a significant cumulative delayed association. Because ambient temperature and relative humidity were strongly inversely correlated at the seasonal level, multicollinearity was carefully evaluated. Variance inflation factor values remained low (1.10–1.47), indicating stable model estimates and suggesting that the observed associations were unlikely to be attributable to collinearity artifacts. Taken together, these findings suggest that humidity-related environmental conditions may be more relevant than ambient temperature alone in explaining short-term variation in pediatric afebrile seizure presentations.

A modest positive association was also observed for precipitation in contemporaneous multivariable models. However, this association was not retained in DLNM analyses, suggesting that precipitation may reflect short-term environmental conditions correlated with humidity rather than an independent delayed meteorological effect. Further studies are needed to clarify the potential role of precipitation and determine whether this finding is reproducible across different climatic regions.

Several biological mechanisms may plausibly underlie an association between elevated humidity and seizure susceptibility. High humidity may impair evaporative heat dissipation and alter thermal perception, potentially increasing physiological stress despite relatively modest ambient temperatures [18,19,20,21,22,23]. Children may be particularly vulnerable because of their distinct thermoregulatory characteristics, including a higher surface area-to-mass ratio than adults. Humidity-associated physiological stress may contribute to autonomic dysregulation, sleep disturbance, electrolyte imbalance, and altered neuronal homeostasis [18,19,20,21,22,23]. Experimental studies have additionally suggested that environmental thermal stress may activate inflammatory pathways and influence cellular microenvironments [24,25]. Although these mechanisms were not directly evaluated in the present study, they provide biologically plausible hypotheses through which meteorological conditions could influence seizure susceptibility.

Alternatively, part of the observed association may reflect meteorologically patterned acute illnesses or other transient physiological conditions rather than a direct effect of humidity on neuronal excitability. Because these potential pathways were not measured at the individual level, the present ecological analysis cannot distinguish direct meteorological effects from indirect, mediated, or residually confounded associations.

Subgroup analyses demonstrated higher point estimates among preschool children, children with established epilepsy, and presentations occurring during warmer months. Although these findings should be interpreted cautiously because of limited statistical power and the absence of formal tests demonstrating effect modification, they raise the possibility that meteorological associations may differ according to age, season, or underlying seizure history. Confirmation of these observations will require larger multicenter studies.

This study has several strengths. Case identification involved a rigorous validation process combining electronic screening with detailed clinical record review, thereby reducing the likelihood of diagnostic misclassification. The combined use of ecological time-series methodology and DLNMs enabled simultaneous assessment of both immediate and delayed meteorological associations. Furthermore, the consistency of findings across multiple sensitivity analyses, including alternative model specifications, supports the robustness of the observed association. Sensitivity analyses using extended 0–5-day and 0–7-day lag windows yielded a similar overall pattern of humidity-associated estimates, and exclusion of frequent presenters did not materially alter the direction or magnitude of the primary association. The availability of a complete meteorological dataset without missing observations further strengthened the exposure assessment.

A deliberate methodological decision was the restriction of the primary environmental exposure analyses to the 2023 dataset. Healthcare utilization patterns during 2019–2022 were substantially influenced by the COVID-19 pandemic and associated public health measures, potentially affecting seizure-related emergency department presentations. Restricting the primary analysis to 2023 provided a more temporally homogeneous framework for evaluating short-term meteorological associations while minimizing the influence of major pandemic-related behavioral and healthcare utilization changes.

Several limitations should also be acknowledged. The retrospective single-center design may limit generalizability to other pediatric populations and climatic settings. Meteorological exposure assessment relied on regional monitoring-station measurements rather than individual-level exposure data. Residual confounding from unmeasured environmental, behavioral, socioeconomic, and clinical factors cannot be excluded. In addition, the relatively small sample size may have limited statistical power, particularly in subgroup analyses. The primary environmental exposure analyses were restricted to a single calendar year, limiting assessment of interannual meteorological variability. Finally, the ecological design precludes causal inference regarding the observed humidity–seizure association.

Meteorological exposures were based on 24-h daily averages rather than measurements preceding the exact time of seizure onset or emergency department presentation. Consequently, same-day exposure estimates for events occurring early in the day may have incorporated meteorological observations occurring after the clinical event, introducing potential temporal exposure misclassification. This limitation is particularly relevant to the interpretation of lag 0 associations and precludes inference regarding the precise temporal sequence between within-day meteorological changes and seizure occurrence. Future studies using hourly or other high-resolution exposure data are needed to establish more precise exposure windows.

Classification of seizure history was based on information available in the institutional electronic medical record and the clinical history documented at presentation. Because this was a single-center retrospective study, previous seizure events or epilepsy diagnoses managed exclusively at other institutions may not have been completely captured. Consequently, some patients with undocumented previous afebrile seizures could have been misclassified as having a first-time presentation. This potential history misclassification should be considered when interpreting the exploratory subgroup analyses according to seizure history.

Although the eligibility criteria excluded several identifiable acute precipitants of seizures, including febrile illness, acute metabolic disturbances, intracranial infections, trauma, toxic exposures, and documented medication non-adherence, other intercurrent illnesses and transient physiological conditions were not systematically measured at the individual level and therefore could not be incorporated as time-varying covariates in the ecological models. Such conditions may themselves vary with meteorological conditions and could potentially confound or mediate part of the observed association between relative humidity and afebrile seizure presentations. Residual confounding by unmeasured acute clinical factors therefore cannot be excluded, and the observed associations should not be interpreted as evidence of a direct causal effect of humidity on seizure occurrence.

Beyond their epidemiological relevance, these findings may have implications for understanding short-term fluctuations in seizure-related healthcare utilization. However, given the single-center ecological design, the one-year environmental exposure period, and the absence of individual-level exposure assessment, the present findings should not be interpreted as supporting humidity-based anticipatory counseling or individual clinical risk prediction. Rather, the observed association should be considered hypothesis-generating and requires confirmation in larger, multicenter, multi-year studies before specific clinical recommendations can be considered. At the population level, further validation of such meteorological patterns may eventually contribute to healthcare resource planning in regions characterized by substantial humidity variability.

## 5. Conclusions

In this ecological time-series study, increased relative humidity emerged as the strongest independent meteorological predictor of pediatric afebrile seizure presentations and was the only environmental exposure demonstrating both significant contemporaneous and cumulative delayed associations. Each 10-percentage-point increase in relative humidity was associated with an 18.6% higher rate of daily pediatric afebrile seizure presentations, and a significant cumulative association persisted across the 0–3-day lag period. In contrast, ambient temperature and precipitation demonstrated less consistent associations across analytical approaches.

These findings suggest that humidity-related environmental conditions may contribute to short-term fluctuations in seizure-related healthcare utilization among children. The observed lag pattern further highlights the importance of considering delayed meteorological associations when investigating relationships between environmental exposures and pediatric afebrile seizure presentations.

From a clinical and public health perspective, the present findings should be interpreted as evidence of a population-level temporal association rather than as a basis for individual risk prediction or humidity-based clinical counseling. Given the single-center ecological design and the one-year environmental exposure period, the findings should be considered hypothesis-generating. If confirmed in larger, multicenter, multi-year studies, meteorological patterns may contribute to understanding short-term variation in seizure-related healthcare utilization and potentially inform healthcare resource planning. Further prospective studies incorporating individual-level and higher-resolution environmental exposure assessment are needed to confirm these findings, evaluate their generalizability across different climatic settings, and clarify the mechanisms underlying the observed humidity-associated pattern.

## Figures and Tables

**Table 1:** Characteristics of the Study Population, Daily Meteorological Conditions, and Pediatric Afebrile Seizure Presentations During the 2023 Study Period.

Variable	Mean ± SD or n (%)	Median (IQR)	Range
Total unique patients	67	—	—
Total seizure presentations	157	—	—
Mean seizure presentations per patient	2.34	—	—
Age of included patients (years)	6.23 ± 4.5	5.3 (2.4–9.1)	0.2–17.0
Female sex	32 (47.9)	—	—
Male sex	35 (52.1)	—	—
Preschool children (<6 years)	39 (58.2)	—	—
School-aged children (≥6 years)	28 (41.8)	—	—
Established epilepsy	40 (59.7)	—	—
First-time afebrile seizure presentation	27 (40.3)	—	—
Daily afebrile seizure presentations (count/day)	0.43 ± 0.71	0 (0–1)	0–4
Days with ≥1 seizure presentation	102 (27.9)	—	—
Seizure-free days	263 (72.1)	—	—
Mean ambient temperature (°C)	18.4 ± 7.1	18.0 (11.2–25.6)	2.1–35.4
Relative humidity (%)	65.5 ± 14.8	63.0 (48.0–76.0)	21.0–96.0
Wind speed (m/s)	2.9 ± 1.0	2.8 (1.9–3.6)	0.8–6.4
Precipitation (mm)	3.8 ± 7.4	0.8 (0.0–4.1)	0.0–72.0
Atmospheric pressure (hPa)	1012.0 ± 7.2	1012 (1007–1017)	998–1029

Table Note: Meteorological variables represent standardized 24-h regional averages obtained from the governmental meteorological monitoring station. Daily afebrile seizure presentations represent pediatric emergency department visits for afebrile seizures recorded per day during the study period. A total of 67 unique patients contributed 157 seizure-related emergency department presentations. Abbreviations: SD, standard deviation; IQR, interquartile range.

**Table 2:** Multivariable Quasi-Poisson Regression Analysis of Meteorological Variables Associated With Daily Pediatric Afebrile Seizure Presentations.

Variable	Adjusted IRR	95% CI	% Change in Presentation Rate	*p*-Value
Mean ambient temperature (+1 °C)	0.972	0.954–0.990	−2.8%	0.003
Relative humidity (+10 percentage points)	1.186	1.103–1.275	+18.6%	<0.001
Atmospheric pressure (+1 hPa)	1.004	0.998–1.010	+0.4%	0.170
Wind speed (+1 m/s)	0.941	0.881–1.006	−5.9%	0.080
Precipitation (+10 mm)	1.115	1.041–1.208	+11.5%	0.002
Weekend vs. weekday	1.062	0.982–1.149	+6.2%	0.130

Table Note: Results were obtained from multivariable Quasi-Poisson regression models adjusted for seasonality, longer-term temporal variation, and day-of-week effects. Incidence rate ratios (IRRs) represent relative changes in the daily rate of pediatric afebrile seizure presentations associated with each predefined exposure increment. For relative humidity, the reported increment represents a 10-percentage-point increase (e.g., from 50% to 60%), rather than a 10% relative increase. Percentage change in presentation rate was calculated as (IRR − 1) × 100. Abbreviations: IRR, incidence rate ratio; CI, confidence interval.

**Table 3:** Cumulative Distributed Lag Non-linear Model Estimates for Meteorological Exposures and Daily Pediatric Afebrile Seizure Presentations.

Exposure	Lag Period (Days)	Cumulative IRR	95% CI	Cumulative % Change	*p*-Value
Relative humidity (+10 percentage points)	0–3	1.15	1.08–1.22	+15.0%	<0.001
Mean ambient temperature (+1 °C)	0–3	0.98	0.91–1.06	−2.0%	0.410
Precipitation (+10 mm)	0–3	1.03	0.98–1.09	+3.0%	0.214

Table Note: Distributed Lag Non-linear Models were used to evaluate cumulative meteorological exposure associations across the predefined lag period of 0–3 days. Cumulative incidence rate ratios represent the overall association between meteorological exposures and the daily rate of pediatric afebrile seizure presentations across the entire lag window after adjustment for seasonality, longer-term temporal variation, and day-of-week effects. Cumulative percentage change was calculated as (IRR − 1) × 100. For relative humidity, the reported increment represents a 10-percentage-point increase (e.g., from 50% to 60%), rather than a 10% relative increase. Relative humidity was the only meteorological exposure demonstrating a statistically significant cumulative association across the 0–3-day lag period. Abbreviations: DLNM, Distributed Lag Non-linear Model; IRR, incidence rate ratio; CI, confidence interval.

## Data Availability

The datasets generated and/or analyzed during the current study are available from the corresponding author on reasonable request. The data are not publicly available because they contain information that could compromise participant privacy and institutional confidentiality.

## Supplementary Materials

The following supporting information can be downloaded at: https://www.mdpi.com/article/10.3390/children13091251/s1, Table S1: Spearman Correlation Matrix Between Daily Pediatric Afebrile Seizure Presentations and Meteorological Variables During the 2023 Study Period; Table S2: Comparison of Meteorological Conditions Between Days With and Without Pediatric Afebrile Seizure Presentations During the 2023 Study Period; Table S3: Sensitivity Analyses of the Association Between Relative Humidity and Daily Pediatric Afebrile Seizure Presentations; Table S4: Exploratory Subgroup Analyses of the Association Between Relative Humidity and Daily Pediatric Afebrile Seizure Presentations; Table S5: Goodness-of-Fit and Dispersion Diagnostics for the Final Multivariable Quasi-Poisson Regression Model; Table S6: Assessment of Multicollinearity Among Meteorological Variables Included in the Primary Multivariable Regression Model.

## Abbreviations

The following abbreviations are used in this manuscript:

AI	Artificial intelligence
CI	Confidence interval
COVID-19	Coronavirus disease 2019
DLNM	Distributed Lag Non-linear Model
GenAI	Generative artificial intelligence
ICD	International Classification of Diseases
ILAE	International League Against Epilepsy
IQR	Interquartile range
IRR	Incidence rate ratio
PACF	Partial autocorrelation function
SD	Standard deviation
TSMS	Turkish State Meteorological Service
VIF	Variance inflation factor
