# Supplementary material for: Short-Term Associations Between Relative Humidity and Pediatric Afebrile Seizure Presentations: A Distributed Lag Non-Linear Time-Series Study
Source: Children (Basel). 2026 Sep 15;13(9):1251. doi: 10.3390/children13091251 (PMC13605689; doi:10.3390/children13091251)
Supplement: Supplementary file 1 [file children-13-01251-s001.zip › children-4509594-supplementary.pdf]

Supplementary Table S1. Spearman Correlation Matrix Between Daily Pediatric Afebrile Seizure Presentations and Meteorological Variables During the 2023 Study Period

Variable	Seizure Counts	Temperature	Relative Humidity	Atmospheric Pressure	Wind Speed	Precipitation
**Seizure counts**	1.00	−0.21**	0.32***	0.09	−0.12	0.18*
**Temperature**	−0.21**	1.00	−0.71***	−0.58***	0.44***	−0.39***
**Relative humidity**	0.32***	−0.71***	1.00	0.66***	−0.41***	0.52***
**Atmospheric pressure**	0.09	−0.58***	0.66***	1.00	−0.55***	0.31***
**Wind speed**	−0.12	0.44***	−0.41***	−0.55***	1.00	−0.22***
**Precipitation**	0.18*	−0.39***	0.52***	0.31***	−0.22***	1.00

Table Note: Values represent Spearman rank correlation coefficients (ρ) calculated using daily observations during the 2023 study period. Correlations between meteorological variables and daily pediatric afebrile seizure presentation counts were considered exploratory and descriptive and were not used as a formal variable-selection procedure for the multivariable models. These unadjusted correlations did not account for seasonality, longer-term temporal variation, or day-of-week effects.

*** $p < 0.05$; ** $p < 0.01$; *** $p < 0.001$.**

Supplementary Table S2. Comparison of Meteorological Conditions Between Days With and Without Pediatric Afebrile Seizure Presentations During the 2023 Study Period

Meteorological Variable	Seizure Days (n = 102), Mean ± SD	Seizure-Free Days (n = 263), Mean ± SD	*p*-value
**Mean ambient temperature (°C)**	16.8 ± 5.4	19.1 ± 6.8	0.041
**Relative humidity (%)**	69.2 ± 13.5	59.3 ± 14.0	<0.001
**Atmospheric pressure (hPa)**	1011.8 ± 7.2	1012.1 ± 7.1	0.620
**Wind speed (m/s)**	2.9 ± 1.1	3.0 ± 1.0	0.270
**Precipitation (mm)**	4.8 ± 8.6	3.4 ± 6.9	0.090

Table Note: Days with at least one eligible pediatric afebrile seizure presentation were classified as seizure days, whereas days without an eligible presentation were classified as seizure-free days. Meteorological variables are presented as mean ± standard deviation (SD). Comparisons between seizure days and seizure-free days were performed using Student's *t*-test or the Mann–Whitney *U* test, as appropriate according to the distribution of the data. These analyses were exploratory and unadjusted and were not used for formal variable selection. Accordingly, the findings should be interpreted alongside the temporally adjusted multivariable Quasi-Poisson and Distributed Lag Non-linear Model analyses.

Abbreviation: SD, standard deviation.

Supplementary Table S3. Sensitivity Analyses of the Association Between Relative Humidity and Daily Pediatric Afebrile Seizure Presentations

Model Specification	IRR per 10-percentage-point increase in RH	95% CI	% Change in Presentation Rate	*p*-value
**Primary multivariable Quasi-Poisson model**	**1.186**	**1.103–1.275**	**+18.6%**	**<0.001**
Standard Poisson model	1.18	1.10–1.26	+18.0%	<0.001
Negative binomial model	1.16	1.08–1.25	+16.0%	<0.001
Univariate humidity model	1.17	1.09–1.25	+17.0%	<0.001

Heatwave-adjusted model	1.14	1.05–1.23	+14.0%	0.002

Table Note: Incidence rate ratios (IRRs) represent relative changes in the daily rate of pediatric afebrile seizure presentations associated with a **10-percentage-point increase in relative humidity** (e.g., from 50% to 60%). The primary multivariable Quasi-Poisson model included mean ambient temperature, relative humidity, atmospheric pressure, wind speed, precipitation, and a binary weekend indicator, with seasonality and longer-term temporal variation controlled using a natural cubic spline for calendar time. Sensitivity analyses evaluated the robustness of the humidity-associated estimate under alternative model specifications. The heatwave-adjusted model additionally included an indicator for days with mean ambient temperature exceeding the 95th percentile of the annual temperature distribution. Percentage change in presentation rate was calculated as (IRR − 1) × 100. Sensitivity analyses were focused on relative humidity because it demonstrated the strongest and most consistent association across the exploratory and primary multivariable analyses.

Abbreviations: CI, confidence interval; IRR, incidence rate ratio; RH, relative humidity.

Supplementary Table S4. Exploratory Subgroup Analyses of the Association Between Relative Humidity and Daily Pediatric Afebrile Seizure Presentations

**Subgroup**	**Subgroup Size**	**IRR per 10-percentage-point increase in RH**	**95% CI**	**% Change in Presentation Rate**	***p*-value**
**Preschool children (<6 years)**	39 patients	1.14	1.06–1.23	+14.0%	<0.001
**School-aged children (≥6 years)**	28 patients	1.07	1.00–1.15	+7.0%	0.048
**Warm season (April–September)**	92 presentations	1.17	1.08–1.27	+17.0%	<0.001
**Cold season (October–March)**	65 presentations	1.05	0.98–1.13	+5.0%	0.110
**Established epilepsy**	40 patients	1.15	1.07–1.24	+15.0%	<0.001
**First-time afebrile seizure presentation**	27 patients	1.04	0.97–1.12	+4.0%	0.210

Table Note: Exploratory subgroup analyses were performed to evaluate the consistency of the association between relative humidity and pediatric afebrile seizure presentations across

demographic, seasonal, and seizure-history categories. Incidence rate ratios (IRRs) represent relative changes in the rate of pediatric afebrile seizure presentations associated with a **10-percentage-point increase in relative humidity** (e.g., from 50% to 60%) within each subgroup. Subgroup analyses were exploratory and were not powered or intended to formally test differences in effect estimates between subgroups; therefore, these findings should not be interpreted as evidence of statistically established effect modification. Percentage change in presentation rate was calculated as **(IRR − 1) × 100**.

Abbreviations: CI, confidence interval; IRR, incidence rate ratio; RH, relative humidity.

Supplementary Table S5. Goodness-of-Fit and Dispersion Diagnostics for the Final Multivariable Quasi-Poisson Regression Mode

Model Diagnostic	Value
**Number of observations**	365
**Residual degrees of freedom**	349
**Residual deviance**	363.0
**Pearson χ^2 statistic**	390.9
**Deviance/df ratio**	1.04
**Pearson χ^2/df ratio**	**1.12**
**Estimated dispersion parameter (ϕ)**	1.12

Table Note: Model fit and dispersion diagnostics were evaluated for the final multivariable Quasi-Poisson regression model. The residual deviance-to-degrees-of-freedom ratio was 1.04, while the Pearson χ^2-to-degrees-of-freedom ratio and estimated dispersion parameter were both approximately 1.12. These findings indicated **modest overdispersion** relative to a standard Poisson model and supported the use of the Quasi-Poisson specification. Residual temporal dependence was evaluated separately using partial autocorrelation function (PACF) plots.

Abbreviations: df, degrees of freedom; PACF, partial autocorrelation function; χ^2, chi-square; ϕ, dispersion parameter.

Supplementary Table S6. Assessment of Multicollinearity Among Meteorological Variables Included in the Primary Multivariable Regression Model

Meteorological Variable	VIF	Tolerance
**Mean ambient temperature**	1.42	0.70
**Relative humidity**	1.28	0.78
**Atmospheric pressure**	1.47	0.68
**Wind speed**	1.18	0.85
**Precipitation**	1.10	0.91

Table Note: Multicollinearity among meteorological predictors included in the primary multivariable regression model was assessed using variance inflation factors (VIFs) and corresponding tolerance values. VIF values >5 were prespecified as indicative of potentially problematic multicollinearity. All observed VIF values were <2.0 (range, 1.10–1.47), and all tolerance values were >0.60 (range, 0.68–0.91), indicating no evidence of problematic multicollinearity among the included meteorological predictors. Although moderate-to-strong pairwise correlations were observed among some meteorological variables in the exploratory Spearman correlation analysis (Supplementary Table S1), these correlations did not translate into problematic multicollinearity in the multivariable model.

Abbreviations: VIF, variance inflation factor
